# A novel class of cysteine protease receptors that mediate lysosomal transport

**DOI:** 10.1111/j.1462-5822.2012.01800.x

**Published:** 2012-05-14

**Authors:** Kumiko Nakada-Tsukui, Kumiko Tsuboi, Atsushi Furukawa, Yoko Yamada, Tomoyoshi Nozaki

**Affiliations:** 1 Department of Parasitology, National Institute of Infectious Diseases1-23-1 Toyama, Shinjuku, Tokyo 162-8640, Japan; 2 Department of Parasitology, Gunma University Graduate School of Medicine3-39-22 Showa-machi, Maebashi 371-8511, Japan; 3 Graduate School of Life and Environmental Sciences, University of Tsukuba1-1-1 Tennoudai, Tsukuba, Ibaraki 305-8572, Japan

## Abstract

The transport of lysosomal proteins is, in general, mediated by mannose 6-phosphate receptors via carbohydrate modifications. Here, we describe a novel class of receptors that regulate the transport of lysosomal hydrolases in the enteric protozoan *Entamoeba histolytica*, which is a good model organism to investigate membrane traffic. A novel 110 kDa cysteine protease (CP) receptor (CP-binding protein family 1, CPBF1) was initially discovered by affinity co-precipitation of the major CP (EhCP-A5), which plays a pivotal role in the pathogenesis of *E. histolytica*. We demonstrated that CPBF1 regulates EhCP-A5 transport from the endoplasmic reticulum to lysosomes and its binding to EhCP-A5 is independent of carbohydrate modifications. Repression of CPBF1 by gene silencing led to the accumulation of the unprocessed form of EhCP-A5 in the non-acidic compartment and the mis-secretion of EhCP-A5, suggesting that CPBF1 is involved in the trafficking and processing of EhCP-A5. The CPBF represents a new class of transporters that bind to lysosomal hydrolases in a carbohydrate-independent fashion and regulate their trafficking, processing and activation and, thus, regulate the physiology and pathogenesis of *E. histolytica*.

## Introduction

Cysteine proteases (CPs) play diverse roles in many aspects of biology and physiology such as growth, development, signalling, programmed cell death and the stress response in both prokaryotes and eukaryotes (Grudkowska and Zagdańska, 2004; Salas *et al*., 2008). While the multi-faceted role of CPs has been revealed, the mechanisms regulating their trafficking in the cell, including specific receptors that determine the committed pathways by which individual CP is sorted to a specific compartment (e.g. lysosomes, plasma membrane, multivesicular bodies and extracellular milieu) remain largely unknown.

In mammalian cells, glycosylated lysosomal hydrolases are transported from the trans-Golgi network (TGN) to endosomes via two types of mannose 6-phosphate (M6P)-specific receptors (MPRs), namely cation-dependent (CD) and independent (CI) MPRs (Ghosh *et al*., 2003; Braulke and Bonifacino, 2009). Both MPRs mediate the segregation of a panel of newly synthesized M6P-containing lysosomal proteins from the secretory pathway. They are known to be involved in the transport of distinct ligands (Qian *et al*., 2008). MPR-dependent traffic is co-ordinately regulated by accessory proteins that interact with the cytosolic portion of MPRs, namely adaptor protein complex-1/2 (AP-1, AP-2) (Höning *et al*., 1997; Ghosh and Kornfeld, 2004), Golgi-associated, gamma-ear containing, ADP ribosylation factor (ARF)-binding protein (GGA) (Puertollano *et al*., 2001; Doray *et al*., 2002), tail-interacting protein of 47 kDa (TIP47) (Díaz and Pfeffer, 1998), phosphofurin acidic cluster-sorting protein 1 (PACS-1) (Crump *et al*., 2001) and retromer (Seaman *et al*., 1998). It is also known that these MPR-associated regulators appear to be engaged in a pathway-specific manner, i.e. TGN to endosomes, the plasma membrane to endosomes, or endosomes to TGN (Jadot *et al*., 1992; Díaz and Pfeffer, 1998; Doray *et al*., 2002; 2007; Arighi *et al*., 2004; Seaman, 2004; Scott *et al*., 2006).

Besides MPR-dependent mechanisms, MPR-independent mechanisms of lysosomal protein trafficking have also been demonstrated in a cell line from I-cell disease patients, in which M6P modifications are absent, and in MPR knockout mice (Dittmer *et al*., 1999; Kornfeld and Sly, 2001). It is conceivable that MPR-independent intracellular targeting is mediated by alternative transportreceptors; however, to date, only two alternative receptors have been identified, sortilin and LIMP-2 (Lefrancois *et al*., 2003; Ni and Morales, 2006; Reczek *et al*., 2007; Canuel *et al*., 2008). Sortilin mediates the lysosomal trafficking of prosaposin, acid sphingomyelinase, cathepsin D and cathepsin H, while LIMP-2 has been identified as a specific receptor for β-glucocerebrosidase.

*Entamoeba histolytica* is the enteric protozoan responsible for amebiasis (Haque *et al*., 2003; Pritt and Clark, 2008). One of the hallmarks of the virulence mechanisms used by this enteric parasite is its ability to invade host tissues by the use of secreted cathepsin-like (Brinen *et al*., 2000) CPs (Que and Reed, 2000; Hellberg *et al*., 2001; Ackers and Mirelman, 2006). While the *E. histolytica* genome contains > 50 CP genes, the majority (> 95%) of CP activity is attributed to four CPs: EhCP-A1, EhCP-A2, EhCP-A5 and EhCP-A7 (Bruchhaus *et al*., 1996; 2003; Loftus *et al*., 2005; Clark *et al*., 2007; Tillack *et al*., 2007; Irmer *et al*., 2009). Among these CPs, EhCP-A1 is not encoded and EhCP-A5 is not expressed in the non-virulent sibling of *E. histolytica*, *E. dispar* (Bruchhaus *et al*., 1996; Willhoeft *et al*., 1999). It has also been shown that repression of EhCP-A5 expression by antisense inhibition caused loss of virulence (Ankri *et al*., 1999). These results strongly suggest the pivotal role of EhCP-A5 in virulence.

Amebic cathepsin-like CPs are also known to be targeted to lysosomes and phagosomes to digest endocytosed fluid and engulfed bacteria and host cells (Marion *et al*., 2005; Mitra *et al*., 2005; Okada *et al*., 2005; 2006). While the *E. histolytica* genome contains genes encoding CD-MPR, AP and retromer, it apparently lacks genes encoding CI-MPR, GGA, TIP47 and PACS-1 (Loftus *et al*., 2005; Nakada-Tsukui *et al*., 2005). Although *E. histolytica* possesses two CD-MPR isotypes (*E*-value: 7.84 × e^−6^ and 4.73 × e^−4^), they do not contain the conserved amino acid motif for M6P binding (Dahms *et al*., 2008). Furthermore, among the four major CPs, EhCP-A1 and EhCP-A2 are not glycosylated (Bruchhaus *et al*., 2003). Despite the fact that EhCP-A5 has a predicted asparagine-linked glycosylation site, it remains unknown whether it is involved in its trafficking (Bruchhaus *et al*., 2003). Therefore, trafficking of CPs in *E. histolytica* likely involves uncharacterized mechanisms.

To date, several molecules that are involved in CP trafficking in *E. histolytica* have been demonstrated. Among nine Rab7 isotypes, at least Rab7A, B, D, E and H are involved in the lysosomal trafficking of CPs, and Rab7A and Rab7B are also involved in the targeting of CPs to phagosomes (Saito-Nakano *et al*., 2004; 2007; Nakada-Tsukui *et al*., 2005). In addition, Rab11B mediates the exocytosis of CPs to the extracellular milieu (Mitra *et al*., 2007). CP trafficking is also regulated by a unique effector, the retromer complex, which likely regulates the retrograde transport of the potential CP receptor by its interaction with Rab7A via a unique carboxyl-terminal extension of Vps26 (Nakada-Tsukui *et al*., 2005). *E. histolytica* also possesses two intrinsic proteinaceous inhibitors of CPs (ICP), a functional homologue of cystatin in mammals (Sato *et al*., 2006). These two ICPs are localized to distinct compartments and negatively regulate the exocytosis/secretion of CPs.

Here we show the identification and characterization of a novel class of transmembrane receptors that specifically interact with and regulate CP trafficking in *E. histolytica*. We designated the putative cysteine protease receptor as CP-binding protein family 1 (CPBF1), which, together with 10 other proteins, consists of a gene family. We examined EhCP-A5 trafficking by using the tetracycline-inducible expression of influenza haemagglutinin (HA)-tagged EhCP-A5, and demonstrated that the repression of CPBF1 causes the enhanced secretion of EhCP-A5 into the extracellular milieu. Time-course kinetic analysis of the localization of HA-tagged EhCP-A5 demonstrated that CPBF1 is involved in the trafficking of EhCP-A5 from the ER to lysosomes.

## Results

### Establishment of a system to monitor EhCP-A5 processing and trafficking

**Fig. 1:**
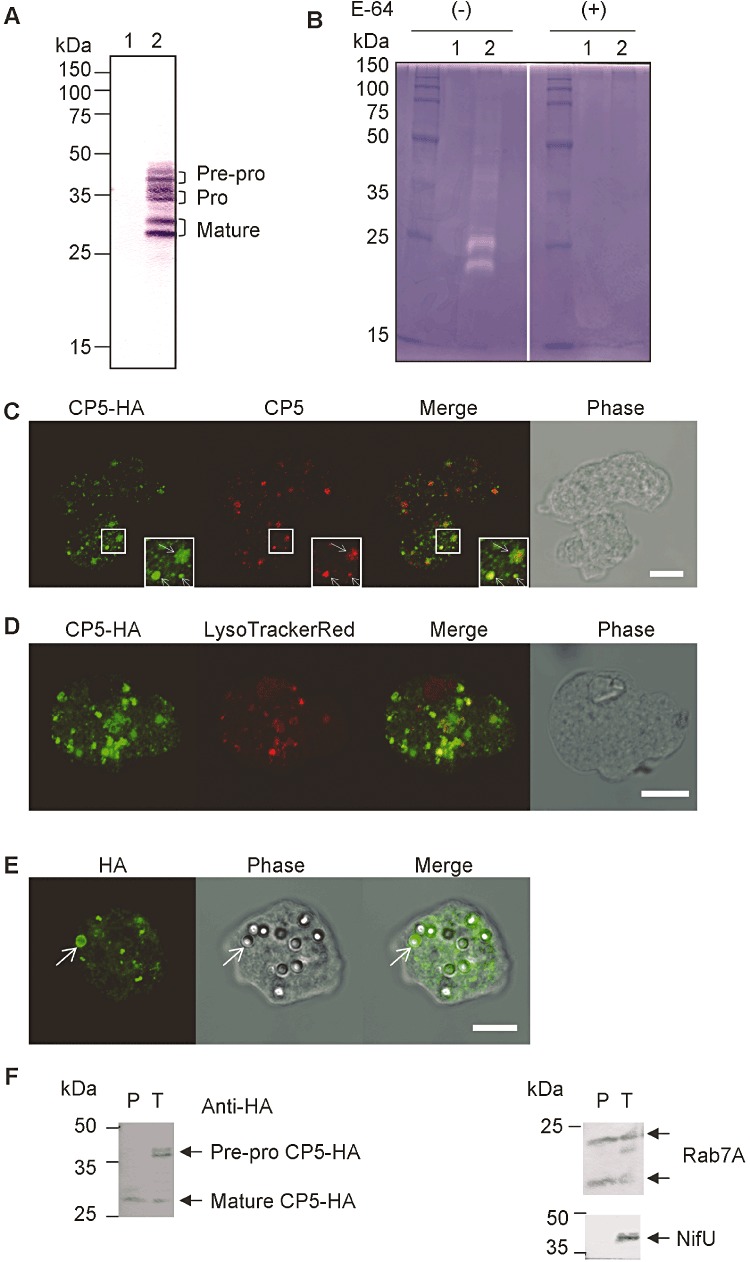
Expression, activity and localization of EhCP-A5-HA. A. Immunoblot analysis of EhCP-A5-HA. Cell lysate from the untransfected (lane 1) or EhCP-A5-HA-expressing (lane 2) *E. histolytica* transformant was analysed by immunoblot analysis with the anti-HA antibody. B. Demonstration of CP activity of EhCP-A5-HA by zymography. Cell lysates from mock-transfected control (lane 1) and the EhCP-A5-HA-expressing transformant (lane 2) were immunoprecipitated with the anti-HA antibody. The immunocomplex was electrophoresed and gelatinolytic activity was visualized in the absence (left) or presence (right) of the CP inhibitor E-64. C. Localization of EhCP-A5-HA. The EhCP-A5-HA-expressing transformant was fixed and reacted with anti-HA (green) and anti-nEhCP-A5 (red) antibodies. Magnified images are shown in the inset panels. Arrows in the inset panels indicates punctate dot-like vesicles of EhCP-A5. Bar, 10 µm. D. Localization of EhCP-A5 in lysosomes. The EhCP-A5-HA-expressing transformant was labelled with LysoTracker Red (red) and reacted with the anti-HA antibody (green). Bar, 10 µm. E. Localization of EhCP-A5 in phagosomes. The EhCP-A5-HA-expressing transformant was incubated with blue fluorescent beads for 16 h, fixed and reacted with the anti-HA antibody. Note that only the phagosome depicted by an arrow is on the confocal plane of the images. Bar, 10 µm. F. Immunoblot analysis of purified phagosomes. Approximately 2 µg of purified phagosomes (P) and total lysate (T) from the EhCP-A5-HA-expressing transformant were subjected to immunoblot analysis using the anti-HA, Rab7A, NifU, EhCP-A1 and EhCP-A5 antibodies.

**Fig. 2:**
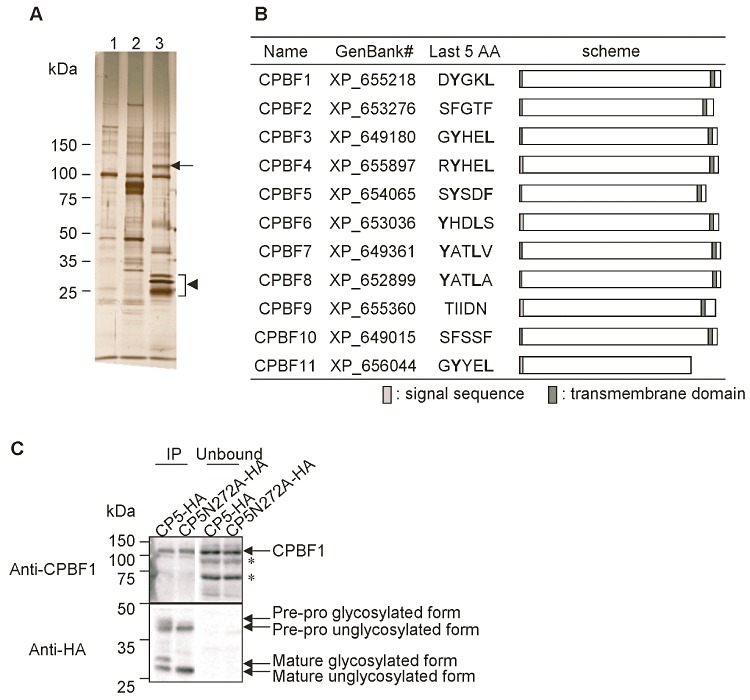
Isolation and identification of an EhCP-A5-binding protein. A. Immunoprecipitation of an EhCP-A5-binding protein. Lysates from the *E. histolytica* transformant expressing HA-Vps35 (lane 2) or EhCP-A5-HA (lane 3) and the control transformant (lane 1) were mixed with the anti-HA conjugated agarose, and the immunocomplex was eluted with the HA peptide as described in *Experimental procedures*. The resultant samples were separated with SDS-PAGE and analysed by silver staining. An arrow indicates cysteine protease-binding protein family 1 (CPBF1). An arrowhead indicates EhCP-A5-HA. B. A schematic diagram of CPBF proteins in *E. histolytica*. Protein names, GenBank accession numbers, peptide sequences of the last five amino acids, and the location of the predicted signal sequence and transmembrane region are shown. Tyrosine and aliphatic amino acids of the YxxΦ motif are indicated in bold. C. Effect of EhCP-A5 deglycosylation on its binding to CPBF1. Lysates from EhCP-A5-HA and EhCP-A5 N272A-HA transformants were immunoprecipitated with the anti-HA antibody and subjected to immunoblot analysis with the anti-CPBF1 or anti-HA antibodies. The asterisks indicate non-specific bands.

**Fig. 3:**
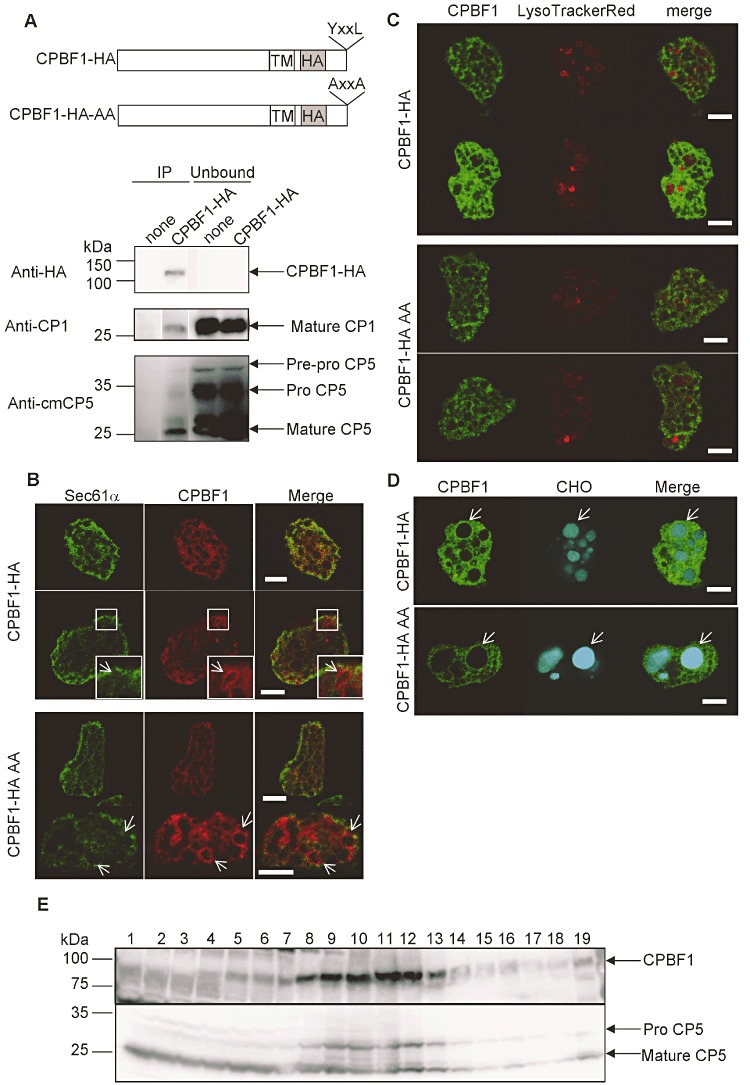
Cellular localization of CPBF1. A. Upper panel, a scheme of epitope-tagged wild-type CPBF1 (CPBF1-HA) and a mutant form of CPBF1 (CPBF1-HA-AA). Lower panel, binding of CPBF1 and CPs. Lysates from CPBF1-HA and control transformant were mixed with the anti-HA antibody and immunoprecipitated (‘IP’), and the unbound fractions were subjected to immunoblot analysis with the anti-HA, anti-EhCP-A1 and anti-cmEhCP-A5 antibodies. The antibodies used are indicated on the left. B. Indirect immunofluorescence assay of CPBF1-HA and CPBF1-HA-AA. CPBF1-HA and CPBF1-HA AA transformants were reacted with the anti-Sec61α (ER marker) and anti-HA antibodies. Bars, 10 µm. The arrows indicate the CPBF1-HA or CPBF1-HA AA-associated vacuoles. Enlarged images are shown in the inset panels. C. Lysosomal localization of CPBF1-HA and CPBF1-HA AA. LysoTracker Red-labelled CPBF1-HA and CPBF1-HA AA transformants were reacted with the anti-HA antibody. Bars, 10 µm. D. Phagosomal localization of CPBF1-HA and CPBF1-HA AA. The CPBF1-HA and CPBF1-HA AA transformant were cultivated with CellTracker Blue-stained CHO cells for 60 min, then fixed and stained with the anti-HA antibody. Bars, 10 µm. The arrows indicate the phagosomes that CPBF1-HA or CPBF1-HA AA are associated with. E. Cellular fractionation of CPBF1 and EhCP-A5. A lysate produced by mechanical homogenization of wild-type trophozoites was separated by discontinuous Percoll gradient ultracentrifugation as described in *Experimental procedures*. Each fraction was subjected to SDS-PAGE and immunoblot analyses using the anti-CPBF1 (upper panel) and anti-cmEhCP-A5 (lower panel) antibodies.

To analyse the trafficking, processing and maturation of EhCP-A5, the major CP implicated in the pathogenesis of *E. histolytica,* we generated an amebic transformant expressing EhCP-A5 fused to the influenza HA epitope at the carboxyl-terminus (EhCP-A5-HA). Immunoblot analysis of the lysate from the EhCP-A5-HA-expressing transformant with an anti-HA antibody showed that EhCP-A5-HA is present in at least 6 distinct forms: the 38 kDa pre-pro-form, which possesses the signal peptide, proregion and mature catalytic domain, the 34 kDa pro-form and the 27 kDa fully processed mature form, and their corresponding glycosylated forms (Fig. 1A). Immunoblot analysis of the parental wild-type amoeba with an anti-cysteine methylated EhCP-A5 (anti-cmEhCP-A5) antibody confirmed that endogenous EhCP-A5 is also detected in the aforementioned multiple forms (see below; Fig. 3A). Asparagine-linked glycosylation was confirmed by immunoblot analysis of the lysates from tunicamycin-treated amoebae; the glycosylated form of the fully processed mature form was abolished by tunicamycin treatment (Fig. S2). Asparagine-linked glycosylation of Asn272 of EhCP-A5 was previously predicted (Bruchhaus *et al*., 2003); thus, we generated an Asn272Ala mutant of EhCP-A5 (EhCP-A5 N272A-HA) and confirmed that this residue is responsible for the glycosylation of EhCP-A5 (Fig. 2C).

Next, to verify that EhCP-A5-HA functions as a protease, its enzymatic activity was examined by gelatin gel electrophoresis (Fig. 1B). EhCP-A5-HA was immunoprecipitated and electrophoresed by sodium dodecyl sulfate-polyacrylamide gel electrophoresis (SDS-PAGE) under non-reducing conditions. E-64-inhibitable CP activity was associated with doublet bands of ∼ 25 kDa in the immunoprecipitate from the EhCP-A5-HA transformant, but not from the control.

### Localization of EhCP-A5

Localization of EhCP-A5 was examined with an immunofluorescence assay (IFA) of the EhCP-A5-HA-expressing transformant using anti-HA and anti-native EhCP-A5 (anti-nEhCP-A5) antibodies (Fig. 1C–E). The distribution of exogenous EhCP-A5-HA detected with the anti-HA antibody showed two distinct localization patterns: punctate dot-like vesicles (Fig. 1C, inset, arrows) and a cytosolic reticular network excluding vesicles. It should be noted that the pattern of EhCP-A5 detected by the anti-nEhCP-A5 antibody differed slightly from the pattern obtained with the anti-HA antibody. The former was confined to vesicles, whereas the latter showed the two different distribution patterns described above. The reticular pattern of EhCP-A5 resembles the broadly distributed reticular network characteristics for *E. histolytica* endoplasmic reticulum (ER) (Sec61α, Fig. 3B; Teixeira and Huston, 2008; Yousuf *et al*., 2010). Altogether, EhCP-A5-HA localized in the reticular network may correspond to the pre-pro- and pro-forms in the ER. A portion of the EhCP-A5-HA vesicles, as detected with the anti-HA antibody, overlapped with LysoTracker Red staining, which accumulates in acidified compartments (Fig. 1D). EhCP-A5-HA was also found in phagosomes that were formed when the trophozoites were co-incubated with carboxylated latex beads (Fig. 1E). Immunoblot analysis of the purified phagosomes showed that the mature processed forms (27 kDa major unglycosylated and 29 kDa minor glycosylated forms) of EhCP-A5-HA were present in phagosomes, whereas the other forms (38 and 40 kDa pre-pro-forms) were also present in the whole lysate (Fig. 1F). These results are consistent with the premise that EhCP-A5-HA mimics the targeting, maturation and function of native EhCP-A5. However, it is also important to note that the processing of EhCP-A5-HA, in particular the cleavage of its signal peptide, is slower than that of native EhCP-A5, which fortuitously allowed further investigation of EhCP-A5 trafficking and processing using this system.

### Identification of an EhCP-A5-binding protein

To better understand the transport mechanism of EhCP-A5, we attempted to isolate a potential transporter that binds to EhCP-A5 using affinity co-immunoprecipitation. Immunoprecipitation of EhCP-A5-binding proteins from the lysate of EhCP-A5-HA-expressing *E. histolytica* trophozoites revealed a specific band of ∼ 110 kDa on SDS-PAGE (Fig. 2A, lane 3). The band was excised and subjected to matrix-assisted laser desorption/ionization time-of-flight mass spectrometry (MALDI-TOF-MS) analysis. The band was identified as a protein of 904 amino acids (a.a.) with a predicted molecular mass of 108 kDa (XP_655218). The same protein was also detected by liquid chromatography-mass spectrometry (LC-MS)/MS analysis of the entire pull-down sample (K. Nakada-Tsukui, unpubl. data). The identified putative EhCP-A5-binding protein contains an amino-terminal signal sequence, a transmembrane domain close to the carboxyl-terminus and a 19-amino-acid-long cytosolic domain containing the YxxΦ motif at the carboxyl-terminus (Fig. 2B, CPBF1). The YxxΦ motif has been shown to interact with the µ-subunit of the adaptor protein (AP) complex (Nakatsu and Ohno, 2003). The AP complex functions at the interphase between a cargo receptor and the clathrin coat, and is often associated with receptors that have a role in trafficking proteins between the TGN and endosomes or between the plasma membrane and lysosomes (Nakatsu and Ohno, 2003; Traub, 2009).

### Genome survey of a novel transmembrane protein family: the cysteine protease-binding protein family (CPBF)

In the *E. histolytica* genome database (http://www.amoebadb.org/amoeba/), 10 additional proteins were identified with similarity (16–23% identity at the amino acid level; *E*-value > 6.30E-21 with blastp) to the identified putative EhCP-A5-binding protein. These proteins have a similar overall size and domain structure, that is, signal peptide, single transmembrane domain and YxxΦ motif (except for three members that do not possess the YxxΦ motif and one that does not possess the transmembrane domain) (Fig. 2B). We named the identified putative EhCP-A5-binding protein as cysteine protease-binding protein family 1 (CPBF1) and the other members as CPBF2–11 in descending order of their similarly to CPBF1. The CPBF is a unique family of proteins with no identifiable homologues in other prokaryotic and eukaryotic organisms.

### Interaction of EhCP-A5 and CPBF1 is mediated by the mature region of EhCP-A5 and is independent of carbohydrates

To confirm the binding of EhCP-A5 and CPBF1 *in vivo*, we generated an *E. histolytica* transformant expressing CPBF1 that contains the HA epitope downstream of the transmembrane region (CPBF1-HA) (Fig. 3A, upper panel). Immunoprecipitation of CPBF1-HA with the anti-HA antibody, followed by immunoblotting with anti-HA, anti-EhCP-A1 and anti-cmEhCP-A5 antibodies, confirmed the *in vivo* binding of CPBF1 and EhCP-A1 and EhCP-A5 (Fig. 3A, lower panel). CPBF1 predominantly bound the mature form of EhCP-A5, compared with the pre-pro- and pro-forms, suggesting that the interaction of CPBF1 and EhCP-A5 occurs via the mature region of CP.

We next examined if the binding of CPBF1 to EhCP-A5 is mediated by carbohydrates, more specifically, the asparagine-linked glycosylation of EhCP-A5 (Fig. 2C). Cell lysates from *E. histolytica* trophozoites expressing EhCP-A5-HA or EhCP-A5 N272A-HA, in which the predicted asparagine-linked glycosylation site (Asn272) was mutated to alanine, were mixed with the anti-HA antibody, and the immunoprecipitates were analysed by immunoblotting with the anti-HA or anti-CPBF1 antibody (Fig. 2C). CPBF1 bound to wild-type and N272A mutant EhCP-A5, suggesting that CPBF1 binds to EhCP-A5 independently of asparagine-linked glycosylation.

We further examined the localization of endogenous CPBF1 by immunoblot assays of cell fractions separated on a Percoll gradient. CPBF1 was fractionated in a similar manner to that of the pro-form of EhCP-A5 (Fig. 3E), but not of its mature form. These data suggest that CPBF1 colocalizes with the pro-form of EhCP-A5 and has a role in the transport of pro-EhCP-A5.

### CPBF1 mainly localizes to the ER, occasionally to phagosomes, but seldom to lysosomes

We examined the localization of HA-tagged CPBF1 (Fig. 3A, upper panel). In the steady state, CPBF1-HA largely overlapped with a subunit of the ER translocon, Sec61α (Fig. 3B, upper panel). In addition, we occasionally found that CPBF1-HA was distributed to the vacuolar membranes without colocalization with Sec61α (Fig. 3B, upper panel, inset) in ∼ 20% of CPBF1-HA transformants (4 out of 23 cells). We further investigated whether CPBF1 is localized to phagosomes (Fig. 3D). CPBF1-HA-expressing transformants were co-cultured with CellTracker Blue-stained Chinese hamster ovary (CHO) cells for 60 min. CPBF1-HA showed phagosomal localization in 7.1% (*n* = 126) of phagosomes. This is consistent with our previous detection of CPBF1 in phagosomes by proteomic analysis (Okada *et al*., 2005; 2006). CPBF1-HA was barely observed on the membrane of lysosomes (Fig. 3C, upper panel). The fact that CPBF1 is rarely localized to phagosomes and lysosomes is consistent with the hypothesis that CPBF1 is involved in the pre-lysosomal/pre-phagosomal trafficking of EhCP-A5.

To understand the importance of the YxxΦ motif in the trafficking of CPBF1, we examined the localization of the CPBF1 mutant CPBF1-HA-AA, in which the tyrosine and lysine residues of the motif were substituted with alanine (Fig. 3A). Unexpectedly, in the steady state and during phagocytosis, the localization of the CPBF1-HA-AA mutant was indistinguishable from that of the wild-type protein (Fig. 3B–D, lower panels). The percentage of cells showing vacuolar or phagosomal localization was 15% (*n* = 26) and 8% (*n* = 138) respectively. We also measured the CP activities in lysates from CPBF1-HA and CPBF1-HA-AA overexpressing transformants. No significant difference in CP activity was detected between these strains and the mock control (K. Nakada-Tsukui, unpubl. data). This indicates that overexpression of the receptor/carrier *per se* does not increase intracellular CP activity. However, we cannot exclude a possibility that we may have overlooked small changes in intracellular CP activity because expression of exogenous CPBF1-HA and CPBF1-HA-AA varied in the transfected population and was confirmed only in 20–40% by immunofluorescence assay.

### Kinetics for the processing and transport of tetracycline-inducible EhCP-A5-HA

**Fig. 4:**
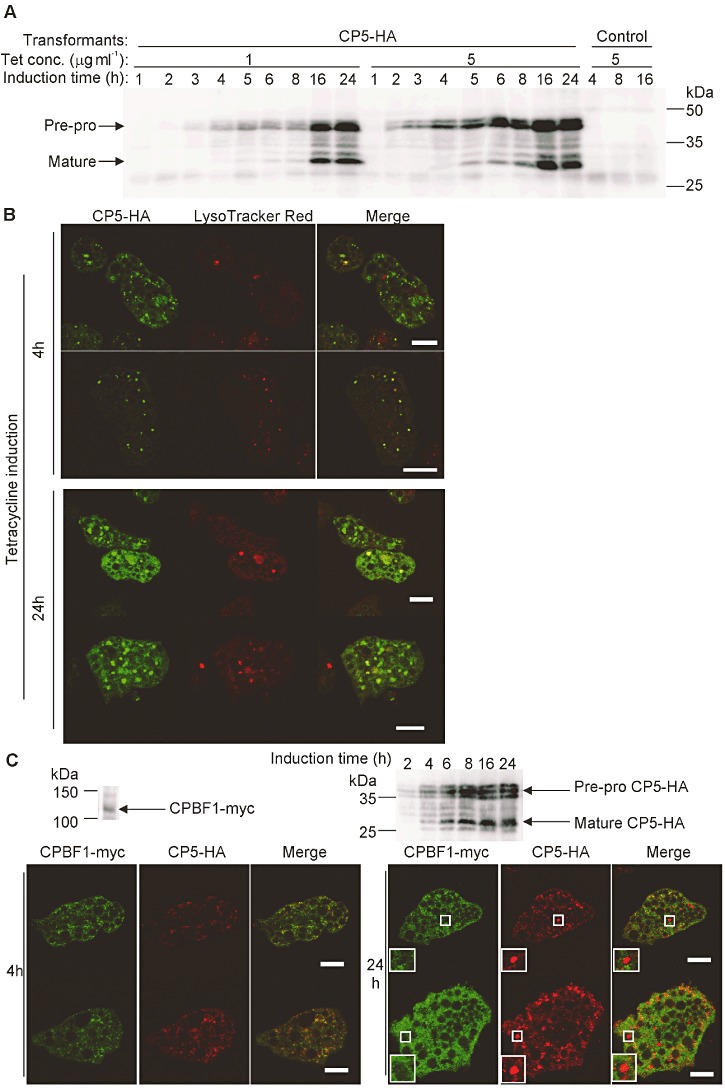
Expression and localization of tetracycline-inducible EhCP-A5-HA. A. Immunoblot analysis of the expression of tetracycline-inducible EhCP-A5-HA. The *E. histolytica* transformant expressing tetracycline-inducible EhCP-A5-HA or the control transformant was cultured in the presence of 1 or 5 µg ml^−1^ tetracycline for the indicated times. The lysates were analysed by immunoblot analysis with the anti-HA antibody. B. Localization of tetracycline-inducible EhCP-A5-HA. The *E. histolytica* transformant expressing tetracycline-inducible EhCP-A5-HA was labelled with LysoTracker Red, further cultured with 5 µg ml^−1^ tetracycline for the indicated times, and subjected to IFA with the anti-HA antibody. Bars, 10 µm. C. Localization of EhCP-A5-HA and CPBF1. The *E. histolytica* line that coexpressed CPBF1-myc constitutively and EhCP-A5-HA in a tetracycline-inducible manner was labelled with LysoTracker Red, further cultured with 5 µg ml^−1^ tetracycline for the indicated times, and subjected to immunoblot analysis (upper panel) or immunofluorescence assay with the rabbit polyclonal anti-HA and mouse monoclonal anti-myc antibodies (lower panel). Magnified images of the area of interest showing EhCP-A5-HA being accumulated within the vesicles that did not colocalize with CPBF1 are shown in rectangles. Bars, 10 µm.

To investigate the time-lapse intracellular trafficking of EhCP-A5, we generated an *E. histolytica* line that expressed EhCP-A5-HA in a tetracycline-inducible manner (tet-EhCP-A5-HA) (Hamann *et al*., 1997; Ramakrishnan *et al*., 1997). The synthesis and processing of EhCP-A5-HA was examined by immunoblot assay at 1–24 h post induction with 1 or 5 µg ml^−1^ tetracycline (Fig. 4A). Induction of EhCP-A5-HA expression with 5 µg ml^−1^ tetracycline predominantly caused the expression of the EhCP-A5-HA pre-pro-forms at 2–8 h, and their subsequent processing into the mature fully processed forms occurred at 16–24 h.

The localization of *de novo* synthesized EhCP-A5-HA was examined by IFA (Fig. 4B). At 4 h, EhCP-A5-HA was detected in the reticular network and the tiny vesicles, the latter of which were partially acidified, as demonstrated by LysoTracker Red staining. At 4 h, no mature EhCP-A5-HA was formed, as demonstrated by immunoblot analysis (Fig. 4A). Therefore, acidification may not be sufficient *per se* for the proteolytic processing of EhCP-A5-HA. At 24 h, when a significant portion of EhCP-A5-HA was present in the fully processed forms (Fig. 4A), EhCP-A5-HA was also distributed to large vacuoles, in addition to the reticular network and tiny vesicles. These data indicate that the large vacuoles contain the fully processed EhCP-A5-HA.

Next, we established an *E. histolytica* line that coexpressed c-myc-tagged CPBF1 (CPBF1-myc) constitutively and EhCP-A5-HA in a tetracycline-inducible manner, and we examined the localization of these proteins at 4 or 24 h post induction with 5 µg ml^−1^ tetracycline (Fig. 4C). At 4 h post induction, CPBF1-myc and EhCP-A5-HA were colocalized in the reticular network. At 24 h post induction, the colocalization of both proteins in the reticular network was still observed; however, a significant proportion of EhCP-A5-HA-positive structures were negative for CPBF1-myc (Fig. 4C). These results are consistent with the premise that EhCP-A5 is synthesized and binds to CPBF1 in the ER and is then translocated to vesicles, where it dissociates from CPBF1.

### Effects of CPBF1 gene silencing on CP activity

**Fig. 5:**
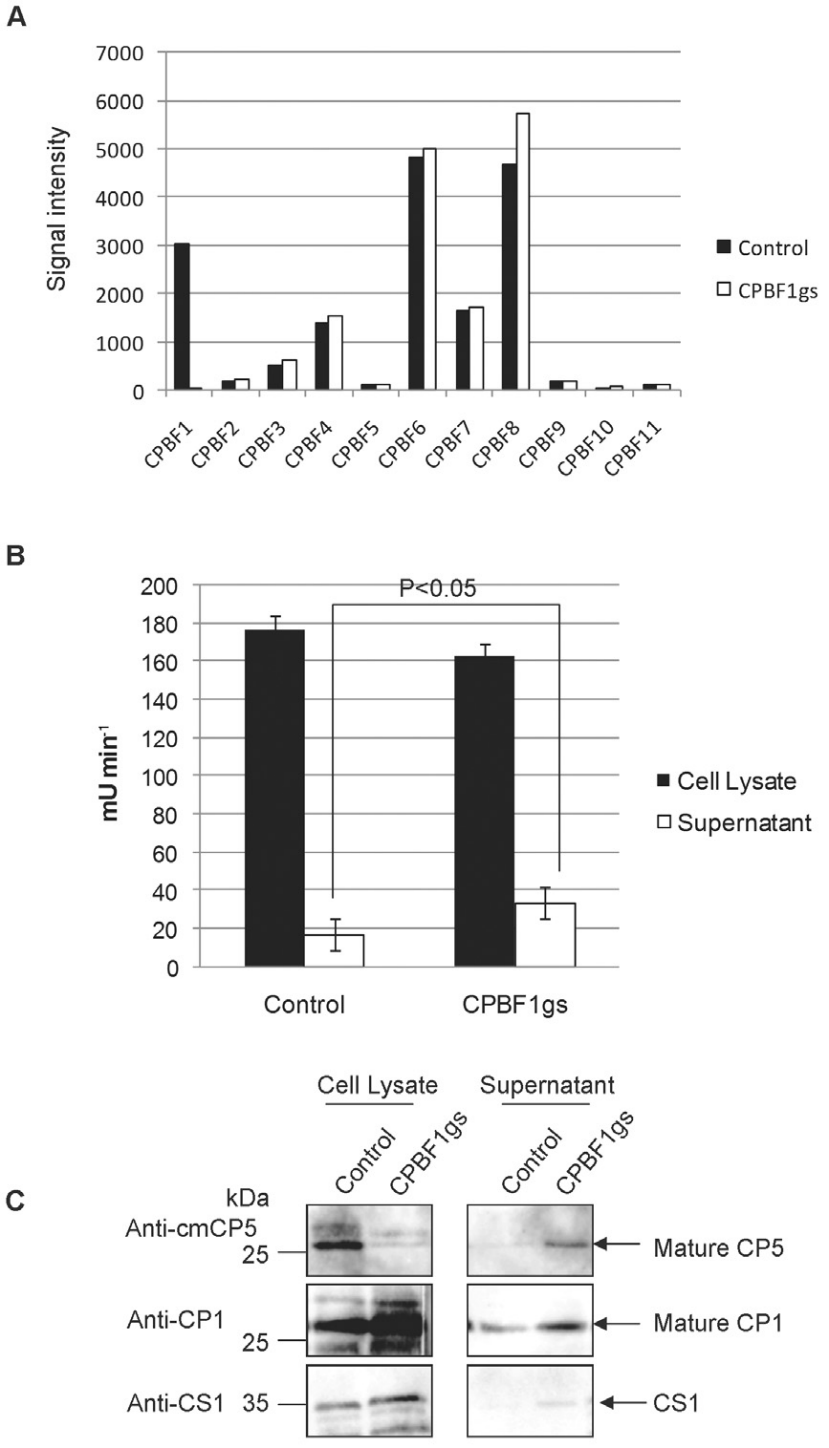
Effect of *CPBF1*- silencing on CPs. A. Expression level of *CPBF* genes in the parental strain (control) and the *CPBF1*-silenced strain (CPBF1gs). RNA from control and CPBF1gs strains was analysed using a custom-made full genome microarray of *E. histolytica* on an Affymetrix platform (Eh_Eia520620F_Eh) (Affymetrix, Santa Clara, CA, USA). B. The effect of *CPBF*-silencing on the activity of intracellular (filled bars) and secreted CPs (open bars). C. Immunoblot analysis of *CPBF1*-silencing on CPs. Approximately 10 µg of lysates from the *CPBF1*-silenced strain (‘CPBF1gs’) or mock (pSAP-2) transformant (‘control’) were separated by SDS-PAGE, and subjected to immunoblot analysis using the anti-cmEhCP-A5, EhCP-A1 and CS1 antibodies. The supernatant from a 2 h culture was concentrated with Amicon Ultra-15 (Millipore, Billerica, MA, USA), and 15 µl of the samples were subjected to SDS-PAGE and immunoblot analyses as described above.

To further investigate the role of CPBF1 in EhCP-A5 trafficking and processing, we generated an amebic transformant strain in which the *CPBF1* gene was repressed by gene silencing (Bracha *et al*., 2006). The gene-specific repression of *CPBF1* was confirmed by transcriptomic analysis (Fig. 5A; whole data were deposited to NCBI Gene Expression Omnibus under Accession No. GSE34368). One-way anova followed by *post hoc* test of three independent experiments showed only four genes were changed with statistical significance: i.e. EHI_164800 (CPBF1), 685-fold downregulation; EHI_179700 (hypothetical protein), 3-fold downregulation; EHI_194510 (DNA topoisomerase putative), 1.4-fold downregulation; EHI_184530 (hypothetical protein), 1.4-fold upregulation. These data reinforce gene-specific repression of CPBF1. We measured the CP activity in the cell and that secreted into the culture supernatant of the *CPBF1*-silenced strain and the parental line. While CP activity in the cell lysate was 8% lower in the *CPBF1*-silenced strain when compared with the mock vector (pSAP2) transfected control strain, CP activity in the culture supernatant of the *CPBF1*-silenced strain was 2.0-fold higher than that of the control strain (Fig. 5B). Immunoblot analysis of the cell lysate and the culture supernatant showed that the amount of intracellular EhCP-A5, but not EhCP-A1 nor cysteine synthase (CS1, used as a cytosolic marker), was remarkably decreased, while the amount of secreted EhCP-A5 was increased (Fig. 5C). The EhCP-A5 mRNA levels remained unchanged, as shown by transcriptomic analysis (9606 versus 10769 in raw fluorescence intensity).

### Processing/maturation and trafficking of EhCP-A5-HA in the CPBF1-silenced strain

**Fig. 6:**
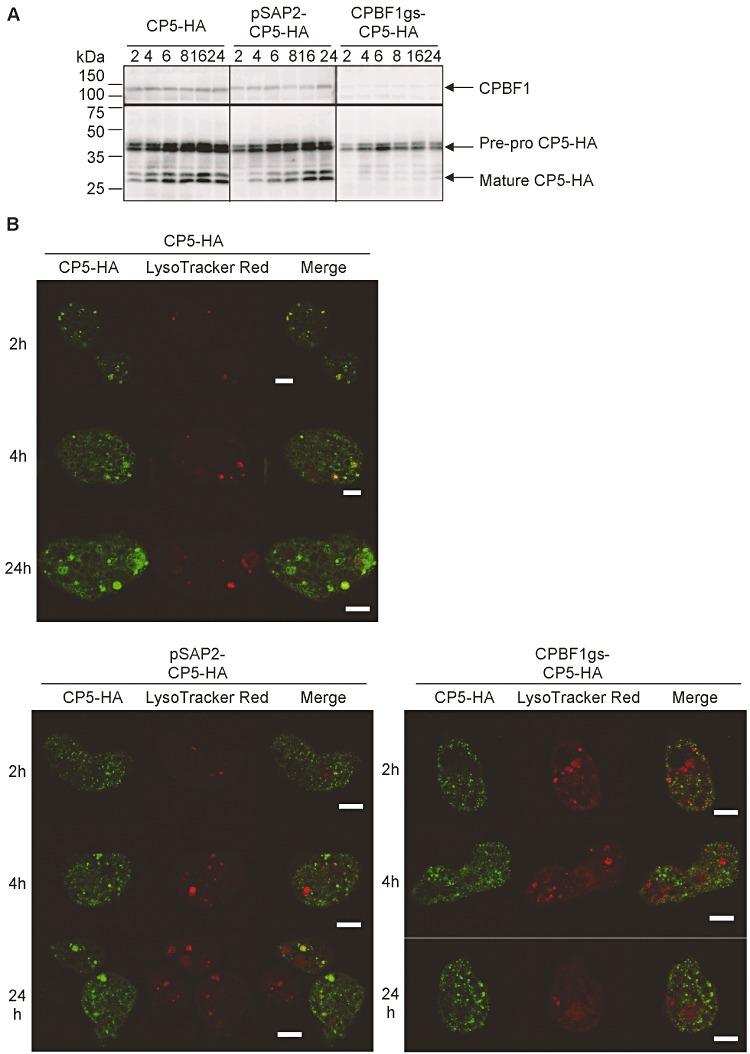
Effect of *CPBF1*-silencing on the processing and distribution of EhCP-A5-HA. A. Immunoblot analysis of lysates from the control G3 strain-originated transformant expressing tetracycline-inducible EhCP-A5-HA (‘EhCP-A5-HA’, left panel), G3-originated transformants double transformed with the pSAP2 control plasmid and tetracycline-inducible EhCP-A5-HA plasmid (‘pSAP2-EhCP-A5-HA’, middle), G3 double transformed with the *CPBF1*-silencing plasmid and tetracycline-inducible EhCP-A5-HA plasmid (‘CPBF1gs-EhCP-A5-HA’, right). The transformants were cultivated with 5 µg ml^−1^ tetracycline for 2–24 h, harvested and subjected to immunoblot analysis using the anti-CPBF1 (top panel) and anti-HA (bottom panel) antibodies. B. Indirect immunofluorescence assay of *CPBF1*-silencing on EhCP-A5 trafficking. The transformants that were labelled with LysoTracker Red were harvested at 2, 4 and 24 h post induction of tetracycline-inducible EhCP-A5-HA, and subjected to IFA using the anti-HA antibody.

We further investigated the role of CPBF1 in the processing, maturation and trafficking of EhCP-A5-HA using a tetracycline-inducible EhCP-A5-HA-expressing G3 strains (Fig. 6). Immunoblot analysis using cell lysates from CPBF1gs-EhCP-A5-HA, in which CPBF1 expression was repressed by gene silencing, showed that EhCP-A5-HA processing was abolished (Fig. 6A, right panel). IFA showed that when CPBF1 was present (i.e. in the EhCP-A5-HA and pSAP2-EhCP-A5-HA strains), EhCP-A5-HA localized to the reticular network and small vesicles at 2 and 4 h post tetracycline induction and to large vacuoles at 24 h post induction. We noted that EhCP-A5-HA-containing vesicles were occasionally acidified (Fig. 6B). On the other hand, in the *CPBF1*-silenced strain, EhCP-A5-HA was observed exclusively in the reticular network, and seldom in either large vacuoles or acidified vesicles, namely lysosomes (Fig. 6B). These results reinforce the hypothesis that CPBF1 is essential for the intracellular trafficking, processing and maturation of EhCP-A5.

The fact that CPBF1 gene silencing resulted in the detection of processed EhCP-A5 (Fig. 5C) and increase in CP activity in the culture supernatant (Fig. 5B) indicates, together with the abolishment of lysosomal transport, processing and activation of EhCP-A5, caused by CPBF1 gene silencing, that EhCP-A5 was mis-secreted as a pre-pro- or pro-form and fortuitously processed extracellularly.

## Discussion

### Discovery of a novel class of receptors essential for the trafficking, processing and maturation of CPs and lysosomal hydrolases

In this study, we described an unprecedented family of hydrolase receptors from a primitive eukaryote, and showed that one member of this family, CPBF1, plays an indispensable role in the trafficking of CPs. There are only two known major classes of CP receptors: M6P-binding receptors (CI-MPR and CD-MPR) and Vps10p/sortilin. Vps10p is a receptor for the vacuolar protease carboxyl peptidase Y in yeast (Marcusson *et al*., 1994). A mammalian Vps10p homologue, sortilin, was shown to be involved in MPR-independent lysosomal trafficking (Lefrancois *et al*., 2003; Ni and Morales, 2006; Canuel *et al*., 2008). CPBF1 is not related to either MPRs or Vps10p/sortilin, according to phylogenetic analysis of the primary amino acid sequences, and thus represents a novel CP receptor that has not been identified in other eukaryotic lineages. While MPR recognizes the phosphorylated mannose residues of CPs, Vps10p binds to a propeptide portion, which functions as a lysosomal targeting signal for carboxyl peptidase Y (Marcusson *et al*., 1994). In contrast, CPBF1 binds to the mature portion of EhCP-A5, and its binding does not depend on carbohydrate modifications. CPBF1 shows no similarity to either the sugar-binding region of MPR or any other known sugar-binding domains; however, CPBF1 shows a low level of similarity (2.3 × e^−3^) to the bacterial pre-peptidase carboxyl-terminal domain (PF04151).

In the *E. histolytica* genome, the CPBF consists of 11 proteins with a similar overall size and domain structure, that is, the signal peptide, a single transmembrane domain and the YxxΦ motif (Fig. 2B). Among these 11 CPBFs, CPBF1, CPBF6 and CPBF8 were previously shown to be localized in phagosomes by proteomic analysis (Okada *et al*., 2005). The related *Entamoeba* species, *E. dispar* and *E. invadens,* also possess 13 and 15 CPBF members respectively. These members were designated based on amino acid similarity and phylogenetic inferences (Table S1, Fig. S1). We identified several lysosomal proteins including amylase, β-hexosaminidase and lysozyme as potential cargo proteins of CPBF6 and 8 (Furukawa *et al*., 2012). These data indicate that the CPBF represents a novel class of hydrolase receptors that have uniquely evolved in this eukaryotic lineage, and play a specific role in the transport of digestive proteins to lysosomes, phagosomes and the extracellular milieu.

### Cargo specificity of CPBF1

Although we identified CPBF1 by virtue of its ability to bind to EhCP-A5, and confirm the interaction with EhCP-A5 and A1 (Fig. 3A), the specificity of CPBF1 towards an array of CPs and other hydrolases is not clear. As our previous phagosome proteomic analysis suggested, at least six CPs, EhCP-A1–6, were transported to phagosomes (Okada *et al*., 2005; 2006). Thus, it is reasonable to presume that CPBF1 binds to more than two CPs. While our pull-down experiment of CPs using epitope-tagged CPBF1 indicate that CPBF1 has affinity to both EhCP-A5 and A1 (Fig. 3A), gene silencing of CPBF1 caused an enhanced secretion of EhCP-A5, but not EhCP-A1 (Fig. 5), suggesting that CPBF1 has a preference towards EhCP-A5. It was previously shown that the knock-down of a hydrolase receptor causes the mis-secretion of their specific ligands (Pohlmann *et al*., 1995; Sohar *et al*., 1998). Our attempt to produce either a full-length or luminal portion of recombinant CPBF1 failed. Thus, the cargo specificity of CPBF1 has not yet been unequivocally proven.

### Localization and binding mechanisms of CPBF1

IFA showed that CPBF1 mainly localizes to the ER (Fig. 3B), and CPBF1 only colocalizes with nascent pre-pro-EhCP-A5-HA (at 4 h of synthesis), and not with mature EhCP-A5-HA in lysosomes (at 24 h) (Figs 3C and 4C). In addition, *CPBF1* gene silencing completely abolished the maturation of EhCP-A5-HA and its transport to lysosomes (Fig. 6). These results indicate that CPBF1 is essential for the transport of EhCP-A5 from the ER to lysosomes.

Although EhCP-A5 was predicted to be glycosylated at Asn272 (Bruchhaus *et al*., 2003), this was proven to be the case *in vivo* for the first time in this study (Figs 2C and S2). To our surprise, this glycosylation is not involved in the trafficking of EhCP-A5 or its interaction with CPBF1 (Figs 2C and S2). Since endogenous EhCP-A5 was also present in the transformant expressing the EhCP-A5-Asn272Ala mutant, there is a possibility that a heterodimer of glycosylated and unglycosylated EhCP-A5 binds to CPBF1. The role of sugar modifications in the activity of EhCP-A5 remains to be determined.

We have shown apparently conflicting evidence for the binding preference of CPBF1 to different forms (i.e. pre-pro-, pro- and mature forms) of EhCP-A5. In the co-immunoprecipitation study, CPBF1 was pulled down predominantly with fully processed mature forms of native EhCP-A5 (Fig. 3A); however, we also showed, using subcellular fractionation, that CPBF1 co-fractionated with the pro-forms of native EhCP-A5 (Fig. 3E). This discrepancy can be explained by the fact that the majority of native EhCP-A5 exists as the mature form with the pre-pro- and pro-forms of native EhCP-A5 remaining in very low amounts, and that processing, particularly of the signal peptide, of the pre-pro-form of EhCP-A5-HA was delayed (or partially hampered) in comparison with native EhCP-A5 (Fig. 1A versus Fig. 3A, unbound). Thus, we propose that CPBF1 is capable of binding to all forms of EhCP-A5 and does not discriminate between the different forms.

Altogether, although CPBF1 is capable of binding to unprocessed and processed forms of EhCP-A5, CPBF1 only interacts, under physiological conditions, with pre-pro- or pro-forms of native EhCP-A5 in the ER, but not with mature EhCP-A5, due to their spatial separation. We also demonstrated that acidification accelerates the dissociation of CPBF1 and EhCP-A5-HA *in vitro*; the interaction between the immunoprecipitated CPBF1/EhCP-A5 complex was disrupted at pH 6 (Fig. S3). This is consistent with the premise that CPBF1 and EhCP-A5 dissociate upon acidification in endosomes/lysosomes, followed by retrograde transport of CPBF1, similar to MPRs (Ghosh *et al*., 2003).

As it is plausible if CPBF1 inhibits CP activity of EhCP-A5 by binding, we measured CP activity, at acidic and neutral pH, of the immunoprecipitated CPBF1-HA/EhCP-A5 complex (K. Nakada-Tsukui, unpubl. data). When the pH of the assay buffer was lowered to pH 6, by which CPBF1 was dissociated from EhCP-A5 (Fig. S3), EhCP-A5 showed a comparable CP activity to that at neutral pH. Thus, the binding of CPBF1 does not appear to inhibit CP activity of EhCP-A5. These data also suggest that the interaction of CPBF1 with EhCP-A5 is mediated by the domain(s) other than the active centre of EhCP-A5. These data are consistent with the observation that CPBF1 apparently binds to the pre-pro- and pro-forms of EhCP-A5 and EhCP-A1 in the ER, where they are inactive.

### Localization of EhCP-A5

Localization of EhCP-A5 demonstrated in this study does not support the inference by the previous study (Jacobs *et al*., 1998), in which EhCP-A5 was identified as a membrane-binding protein and localized to the plasma membrane by transmission electron microscopy using an antibody raised against 12-a.a.-long peptide specific to EhCP-A5. In the present study, we used both anti-HA and anti-nEhCP-A5 recombinant antibodies for immunofluorescence assay. The discrepancy of localization of EhCP-A5 between the studies may indicate a few possibilities: first, the anti-peptide antibody recognizes the plasma membrane-localized form better for unknown reasons; second, EhCP-A5 may be recruited to the plasma membrane under certain conditions we have not yet determined, e.g. nutrients, oxygen tension, growth factors and chemoattractants; third, localization of EhCP-A5 may be different between the laboratory strains of HM-1:IMSS. Immunofluorescence assay using live cells at 4°C did not demonstrate surface staining (K. Nakada-Tsukui, unpubl. data). However, it is also worth noting that we occasionally observed membrane localization of EhCP-A5-HA on vesicles in the strain where EhCP-A5-HA was constitutively expressed or when we induced expression of EhCP-A5-HA with tetracycline for 16 or 24 h (Fig. 4B and K. Nakada-Tsukui unpubl. data). Together with the fact that we detected membrane-associated EhCP-A5 from the organelle fraction of 100 000 *g* ultracentrifugation (K. Nakada-Tsukui, unpubl. data), membrane association/recruitment of EhCP-A5 probably occurs, but detailed localization, i.e. the plasma membrane or internal membrane, needs to be determined in the future.

### The role of the YxxΦ motif on the localization of CPBF1

The cytosolic domain of CPBF proteins was structurally similar: it consists of 11–38 a.a. and is terminated with the YxxΦ motif in 9 out of 11 members. In CPBF1, the motif in the 19-a.a. cytosolic domain is involved in its interaction with other accessory protein(s) and regulates CPBF1 trafficking. However, unexpectedly, the localization of CPBF1 was not altered by mutation of the YxxΦ motif (Fig. 3), although the *E. histolytica* genome encodes major components of AP complexes and clathrines (Loftus *et al*., 2005; Clark *et al*., 2007). Although we cannot exclude the possibility that mutation of the motif caused unnoticeable minor changes in localization, we tentatively conclude that the presence of the motif in CPBF1 does not primarily determine its localization.

We also examined binding between CPBF1 and a panel of proteins that are known to be involved in CP transport; however, CPBF1 did not show a significant interaction with EhVps26, EhVps35, EhRab7A, EhRab11B or ICP1 (K. Nakada-Tsukui, unpubl. data). These proteins are likely involved in the later steps of CP transport/secretion, but not in its transport from the ER.

In summary, we described a novel family of hydrolase receptors in eukaryotes. We propose that CPBF1 plays an indispensable role in the trafficking and activation of CPs, which are important virulence factors of *E. histolytica*. This is the first report of CP receptor/carriers in pathogenic eukaryotes and, as such, should help to reveal the underlying pathogenicity of microbial pathogens.

## Experimental procedures

### Cells and reagents

Trophozoites of *E. histolytica* strain HM-1:IMSS cl6 (HM-1) (Diamond *et al*., 1972) and strain G3 were maintained axenically in Diamond's BI-S-33 medium (Diamond *et al*., 1978) at 35.5°C. Amoeba transformants were cultured in the presence of 10 or 40 µg ml^−1^ Geneticin or 4 µg ml^−1^ hygromycin (Invitrogen, San Diego, CA, USA). CHO cells were maintained in F12 medium (Invitrogen, San Diego, CA, USA) supplemented with 10% fetal calf serum (MBL, Nagoya, Japan) at 37°C with 5% CO_2_. *Escherichia coli* strain DH5α was purchased from Life Technologies (Tokyo, Japan). All chemicals of analytical grade were purchased from Sigma-Aldrich (Tokyo, Japan) unless otherwise stated.

### Plasmid construction

Standard techniques were used for routine DNA manipulation, subcloning and plasmid construction (Sambrook and Russell, 2001). To produce EhCP-A5-HA expression vectors, the full-length protein coding region of EhCP-A5 without the stop codon was amplified by PCR with primers containing a BglII site at the 5′ end and a XhoI site at the 3′ end, and subcloned into a BglII and XhoI-double-digested pEhEx vector (Hamann *et al*., 1995). The resultant plasmids were designated as pEhEx-EhCP-A5. A self-annealing pair of oligonucleotides corresponding to the HA peptide containing SalI and XhoI sites at the end were inserted into the XhoI site of pEhEx-EhCP-A5 to produce pEhEx-EhCP-A5-HA. The tetracycline-inducible EhCP-A5-HA expression vector (ptet-EhCP-A5-HA) was generated by subcloning the protein coding region of EhCP-A5-HA PCR-amplified from pEhEx-EhCP-A5-HA with primers containing KpnI and BamHI sites, into a KpnI- and BamHI-double-digested pEhHygtetR-O-Cass vector, a kind gift from Dr William A. Petri Jr (Hamann *et al*., 1997; Ramakrishnan *et al*., 1997). The plasmid to express a mutant form of EhCP-A5-HA containing the N272A substitution (pEhEx-EhCP-A5 N272A-HA) was generated by PCR-based site-directed mutagenesis. To produce the CPBF1-HA expression vector, two fragments corresponding to a.a. 1–890 and 886–904 of the CPBF1 protein coding region were PCR-amplified with primers containing a BamHI site or SmaI and XhoI sites, respectively, and sequentially subcloned into the BglII site and SmaI/XhoI sites of pEhExHA, respectively, to produce pEhExCPBF1-HA (Nakada-Tsukui *et al*., 2009). The plasmid to express a mutant form of CPBF1-HA that possesses two alanine substitutes in the YxxΦ motif (pEhExCPBF1-HA-AA) was generated by PCR-based site-directed mutagenesis. To express the c-myc-tagged proteins, vectors were constructed which allowed to express a protein of interest with three tandem copies of the c-myc (Myc) epitopes, this plasmids were designated as pEhExMyc. pEhExMyc vector was generated by the insertion of annealed oligonucleotides corresponding three tandem copies of the Myc epitope flanked by 5′ BglII site and 3′ SmaI and XhoI sites into the BglII–XhoI site of pEhEx (Hamann *et al*., 1995).To produce the CPBF1-myc expression vector, full length of the CPBF1 protein coding region were PCR-amplified with primers containing a BamHI site and subcloned into the BglII site of pEhExMyc vector (pEhExMyc-CPBF1). To generate the plasmid to produce an amoeba line in which CPBF1 expression is repressed, the 310 bp region corresponding to the amino-terminus of the CPBF1 protein coding region was PCR-amplified and subcloned into the StuI and SacI sites of pSAP2-Gunma (Mi-ichi *et al*., 2011; Penuliar *et al*., 2011). To generate the plasmid to produce histidine-tagged CPBF1 recombinant protein, a coding region corresponding to a.a. 638–860 was PCR-amplified and cloned into the StuI and HindIII sites of pET47b (Novagen, Madison, WI, USA).

### Antibodies

The anti-HA (clone 11MO) and anti-myc (clone 9E10) monoclonal antibodies were purchased from Covance (Princeton, NJ, USA). The anti-HA rabbit polyclonal antibody was purchased from BML (Nagoya, Japan). The production of rabbit polyclonal antibodies against denatured EhCP-A1, EhCP-A5, Vps26, Rab7A, and nitrogen fixation U was previously described (Ali *et al*., 2004; Saito-Nakano *et al*., 2004; Nakada-Tsukui *et al*., 2005; Mitra *et al*., 2007). The anti-Sec61α and anti-dolichol-*P*-mannose synthase (DPMS) rabbit polyclonal antibodies (Salgado *et al*., 2005) were a kind gift from Dr Rosana Sánchez-López. The anti-native EhCP-A5 (nEhCP-A5) antibody used for IFA was a kind gift from Dr Sharon Reed. The rabbit antibody against cysteine-methylated EhCP-A5 (cmEhCP-A5) was commercially produced against histidine-tagged EhCP-A5 recombinant protein and generated as follows. The mature form of histidine-tagged EhCP-A5 was purified in denaturing conditions as previously described (Sato *et al*., 2006). Recombinant EhCP-A5 protein was dialysed against dialysis buffer (0.5 M Tris-HCl pH 8.5, 8 M urea, 10 mM EDTA) for 16 h at 4°C, and then incubated with 25 mM dithiothreitol for 1 h at room temperature. The mixture was further incubated with 75 mM iodoacetamide for 1 h at room temperature in the dark. The histidine-tagged cmEhCP-A5 recombinant protein was dialysed against dialysis buffer for 16 h at room temperature, and used to immunize rabbits. The anti-cmEhCP-A5 antibody recognized the mature form of endogenous EhCP-A5 as well as the pre-pro- and pro-forms of EhCP-A5, whereas the anti-EhCP-A5 antibody preferentially reacted against the mature form of EhCP-A5. The rabbit antibody against CPBF1 was commercially raised against recombinant histidine-tagged CPBF1 partial protein (a.a. 638–860) and was produced and purified as follows. The expression plasmid for histidine-tagged CPBF1 (638–860) was introduced into BL21(DE3) competent cells (Invitrogen, San Diego, CA, USA). Expression of the recombinant protein was induced with 0.1 mM isopropyl-β-thiogalactoside at 37°C for 3 h. The histidine-tagged fusion protein was purified under denaturing condition using Ni-NTA agarose (QIAGEN, Hiden, Germany), according to the manufacturer's instructions.

### Amoeba transformation

The plasmids generated as described above were introduced into HM-1 trophozoites by lipofection, as previously described (Nozaki *et al*., 1999). To generate double transformant which express CPBF1-myc and tet-EhCP-A5-HA, pEhExMyc-CPBF1 plasmid was transfected to tet-EhCP-A5-HA expressing strain then maintained in the presence of 4 µg ml^−1^ hygromycin and 10 µg ml^−1^ G418. For the generation of the *CPBF1*-silenced strain, plasmids, pSAP2-Gunma-CPBF1 and pSAP2-Gunma, as a control, were introduced into the trophozoites of the *E. histolytica* G3 strain, which was a kind gift from Dr David Mirelman. Geneticin or hygromycin was added at a concentration of 1 µg ml^−1^ at 24 h after transfection, and gradually increased for ∼ 2 weeks until the G418 concentration reached 10 µg ml^−1^ for the EhCP-A5-HA, CPBF1-HA, pSAP2 and pSAP2-CPBF1 transformants or 6 µg ml^−1^ for the pSAP2+ptetEhCP-A5-HA and pSAP2-CPBF1+ptetEhCP-A5-HA transformants respectively. The hygromycin concentration was also increased until it reached 4 µg ml^−1^ for the tet-EhCP-A5-HA transformant. To create double transformants, after the *CPBF1*-silenced strain was established, they were further cultivated without G418 for at least 4 weeks to remove the silencing plasmids, and G418 sensitivity was examined. The *CPBF1*-silenced strain and mock transformant strain were then transfected with ptet-EhCP-A5-HA and maintained in the presence of 10 µg ml^−1^ hygromycin.

### Indirect immunofluorescence assay

The indirect immunofluorescence assay was performed as previously described (Saito-Nakano *et al*., 2004) with slight modifications. Briefly, the amoeba transformant cells were harvested and transferred to 8 mm round wells on a slide glass, and then fixed with 3.7% paraformaldehyde in phosphate-buffered saline (PBS), pH 7.2, for 10 min. After washing, the cells were permeabilized with 0.2% saponin in PBS containing 10% bovine serum albumin (BSA) for 10 min, and reacted with a primary antibody diluted at 1:30 (anti-Sec61α, anti-DPMS, anti-nEhCP-A5 and anti-myc antibodies) or 1:1000 (anti-HA monoclonal and anti-HA polyclonal antibodies) in PBS containing 0.2% saponin and 10% BSA. After washing, the samples were then reacted with Alexa Fluor 488- or 568-conjugated anti-rabbit or anti-mouse secondary antibody (1:1000) for 1 h. For phagosome staining, CHO cells were pre-stained with 10 µM CellTracker Blue (Molecular Probes, Eugene, OR, USA) for 30 min, harvested and washed with BI-S-33 medium. Approximately 1.5 × 10^5^ amoeba cells were incubated with 3 × 10^5^ of CellTracker-stained CHO cells for the indicated times. For lysosomal staining, 10 µM LysoTracker Red (Molecular Probes, Eugene, OR, USA) was added to *E. histolytica* transformants for 16 h, and the trophozoites were then washed, harvested and subjected to IFA. To visualize phagosomes, EhCP-A5-HA-expressing transformant trophozoites were co-cultured with blue fluorescent carboxylate-modified beads (Molecular Probes, Eugene, OR, USA) for 16 h, harvested and subjected to IFA. The samples were examined on a Carl-Zeiss LSM 510 META confocal laser-scanning microscope. The resultant images were further analysed using LSM510 software.

### Immunoprecipitation

For the isolation of EhCP-A5-HA-binding proteins, the cell pellet from 1.0 × 10^7^ EhCP-A5-HA, HA-Vps35 or wild-type HM-1-expressing cells was lysed with 1 ml of lysis buffer [50 mM Tris-HCl pH 7.5, 150 mM NaCl, 1% Triton X-100, 0.5 mg ml^−1^ E-64, complete mini EDTA-free protease inhibitor cocktail (Roche Applied Science, Penzberg, Germany)]. The soluble lysate, after centrifugation at 14 000 *g*, was pre-cleared with 50 µl of protein G Sepharose (GE Health Care, Waukesha, WI, USA) and then mixed and incubated with 90 µl of anti-HA monoclonal antibody-conjugated agarose (Sigma-Aldrich, St. Louis, MO, USA) for 3.5 h at 4°C. Immune complexes bound to the resin were washed three times with wash buffer (50 mM Tris-HCl pH 7.5, 150 mM NaCl, 1% Triton X-100) and then eluted by incubating the resin with 180 µl of 100 mg ml^−1^ HA peptide (Sigma-Aldrich, St. Louis, MO, USA) in lysis buffer for 16 h at 4°C. Eluted samples (2 µg) were subjected to SDS-PAGE and visualized with a silver stain MS kit (WAKO, Tokyo, Japan). The specific 110 kDa band was excised and subjected to MS. For co-immunoprecipitation analysis, lysates of the amoeba transformants expressing EhCP-A5-HA, EhCP-A5 N272A-HA, CPBF1-HA or non-transfected HM-1 were pre-cleared with protein G Sepharose. Protein G Sepharose was pre-mixed with an anti-HA monoclonal antibody (11MO) (Covance, Princeton, NJ, USA) in the presence of 10 mg ml^−1^ BSA for 1 h at 4°C, and unbound antibody was removed by washing the resin with lysis buffer. The cleared lysates and antibody-bound protein G Sepharose were co-incubated for 2 h at 4°C, and immune complexes bound to the resin were subjected to SDS-PAGE and immunoblot analyses as previously described (Sambrook and Russell, 2001). Primary antibodies were used at a 1:100 dilution for anti-EhCP-A1, EhCP-A5, Cm-EhCP-A5, CPBF1 and Rab7A, or at a 1:1000 dilution for anti-nitrogen fixation U and HA in immunoblot analyses.

### Gelatin gel electrophoresis

The protease activity of HA-tagged EhCP-A5 was assayed by gelatin substrate gel electrophoresis as described previously (Reed *et al*., 1989; Hellberg *et al*., 2002). Briefly, the immune complex was pulled down from untransfected or EhCP-A5-HA-expressing amoebae with the anti-HA antibody as described above, except that 1 mM dithiodipyridine was used instead of E-64 and complete mini EDTA-free protease inhibitor cocktail. The immunoprecipitated sample was electrophoresed under non-reducing conditions on a 12% SDS-polyacrylamide gel copolymerized with 0.1% gelatin (Sigma-Aldrich, Tokyo, Japan). After separation, the gel was washed with 2.5% Triton X-100 for 1 h to remove SDS, and incubated in the reaction buffer with or without 0.5 mg ml^−1^ E-64 (100 mM sodium acetate pH 4.0, 1% Triton X-100, 2 mM dithiothreitol) at 37°C for 5 h. The gels were stained with Coomassie Brilliant Blue.

### Mass spectrometric analysis

MALDI-TOF-MS analysis of the excised 110 kDa band was performed by the Hitachi High-Technologies Corporation (Tokyo, Japan). Briefly, the excised 110 kDa band was digested with trypsin and subjected to peptide mass fingerprinting using the MALDI-Qq-TOF MS/MS QSTAR Pulsar *i* system (Applied Biosystems, Foster City, CA, USA). The acquired data were searched against the NCBI nr database by MASCOT software (Matrix Science, Boston, MA, USA). The immune complex obtained from EhCP-A5-HA and HA-Vps35-expressing transformants and non-transfected HM-1 were subjected to trypsin digestion and analysed on a LC-MS system consisting of a Finnigan LCQ ion trap mass spectrometer system with a Protana nanospray ion source interfaced to a self-packed Phenomenex Jupiter 10 mm C18 reversed-phase capillary column (8 cm by 75 mm) at the W. M. Keck Biomedical Mass Spectrometry Laboratory, University of Virginia. The sequencing data were analysed against the *E*. *histolytica* genome database (http://pathema.jcvi.org/cgi-bin/Entamoeba/PathemaHomePage.cgi) at the J. Craig Venter Institute using the Sequest algorithm (Eng *et al*., 1994) and also against the NCBI nr database.

### Microarray analysis of the CPBF1-silenced strain

The expression profiles of all the genes in *E. histolytica* were analysed using a custom-made full genome microarray of *E. histolytica* on an Affymetrix platform. The array contained 9326 independent probe sets, each of which had 11 probe pairs. The probes were designed according to the genome sequence of the *E. histolytica* reference strain HM-1, available from the Pathema Bioinformatics Resource Center (data release 6.0). RNA was isolated and purified from G3 strains transfected with pSAP2-Gunma or pSAP2-Gunma-CPBF1 using the TRIzol reagent (Invitrogen, San Diego, CA, USA). The quality of the RNA was examined with the Experion system (Bio-Rad, Hercules, CA, USA). Approximately 5 µg of RNA was reverse transcribed to cDNA and used to synthesize biotin-labelled cRNA. After purification, cRNA was fragmented and hybridized onto a custom-made probe array chip (Eh_Eia520620F; Affymetrix, Santa Clara, CA, USA). Following hybridization, the arrays were washed and stained with streptavidin-phycoerythrin (Molecular Probes, Eugene, OR, USA) using an Affymetrix GeneChip Fluidics Station 450. Arrays were scanned with an Affymetrix GeneChip Scanner 3000 at 570 nm (Husain *et al*., 2011). Data were analysed with GeneSpring software by Tohoku-Kagaku-Yakuhin (Iwate, Japan). The whole data set was deposited in NCBI Gene Expression Omnibus (Edgar *et al*., 2002) (Accession No. GSE34368; http://www.ncbi.nlm.nih.gov/geo/).

### CP assay

CP activity was measured with the cleavage of the synthetic peptide substrate z-Arg-Arg-7-amino-4-trifluoromethylcoumarin (ICN, Aurora, OH, USA) as described previously (Nakada-Tsukui *et al*., 2005). Activity was expressed in mmol of 7-amino-4-trifluoromethylcoumarin produced per mg of lysate protein. The statistical significance of the data was evaluated with Student's *t*-test.

### Phagosome preparation

Phagosomes were prepared as previously described (Okada *et al*., 2005; 2006). The isolated phagosomes were subjected to immunoblot analyses.

### Cell fractionation

Cell fractionation was performed as previously described (Mi-ichi *et al*., 2009). Each fraction was analysed by immunoblotting with anti-CPBF1 and anti-CmEhCP-A5 antibodies.

## Supporting information

**Table S1.** CPBFs from *E. dispar* and *E. invadens*. The *E. dispar* and *E. invadens* genome database (http://pathema.jcvi.org/cgi-bin/Entamoeba/PathemaHomePage.cgi) was searched using *E. histolytica* CPBFs as query sequences. All potential *E. dispar* and *E. invadens* CPBFs were retrieved and analysed with all *E. histolytica* CPBFs (Fig. S1). *E. dispar* and *E. invadens* CPBFs were annotated based on phylogenetic inferences and their percentage identity to the corresponding *E. histolytica* homologues.

**Fig. S1.** Phylogenetic analysis of CPBFs from *E. histolytica*, *E. dispar* and *E. invadens*.Phylogenetic reconstruction was performed using CLUSTAL W. Trees were drawn using CLUSTAL W with p-distance method. The numbers at the nodes represent the bootstrap values for 1000 iterations. The scale bar indicates 0.1 substitutions at each amino acidposition.

**Fig. S2.** Verification of the sugar modification of EhCP-A5 by tunicamycin treatment. *E. histolytica*trophozoites were treated with 0, 3 or 10 μg ml^−1^ tunicamycin for 24 h. Cells were harvested, lysed and analysed by immunoblot analysis with anti-EhCP-A5, anti-EhCP-A1 and anti-Hgl antibodies. Note that the apparent molecular weight of Hgl decreased, indicating removal of a sugar moiety by tunicamycin treatment. Also note that the intensity of the upper glycosylated form of mature EhCP-A5 decreased in a dose-dependent manner.

**Fig. S3.** Dissociation of EhCP-A5 and CPBF1. CPBF1-HA-expressing *E. histolytica* trophozoites were subjected to immunoprecipitation with anti-HA antibody as described in *Experimental procedures*. Precipitated samples were further incubated in 90 μl of phosphate/citric acid buffer, pH7.0 or pH 6.0, for 3 min, and centrifuged at 1000 *g* for 1 min to separate supernatant (‘sup’) and agarose bead (‘beads’) fractions. The samples were analysed by immunoblot analysis with anti-CPBF1 or anti-EhCP-A5 antibody.
