# Supplementary figures and images for: A novel class of cysteine protease receptors that mediate lysosomal transport
Source: Cell Microbiol. 2012 May 14;14(8):1299–317. doi: 10.1111/j.1462-5822.2012.01800.x (PMC3465781; doi:10.1111/j.1462-5822.2012.01800.x)

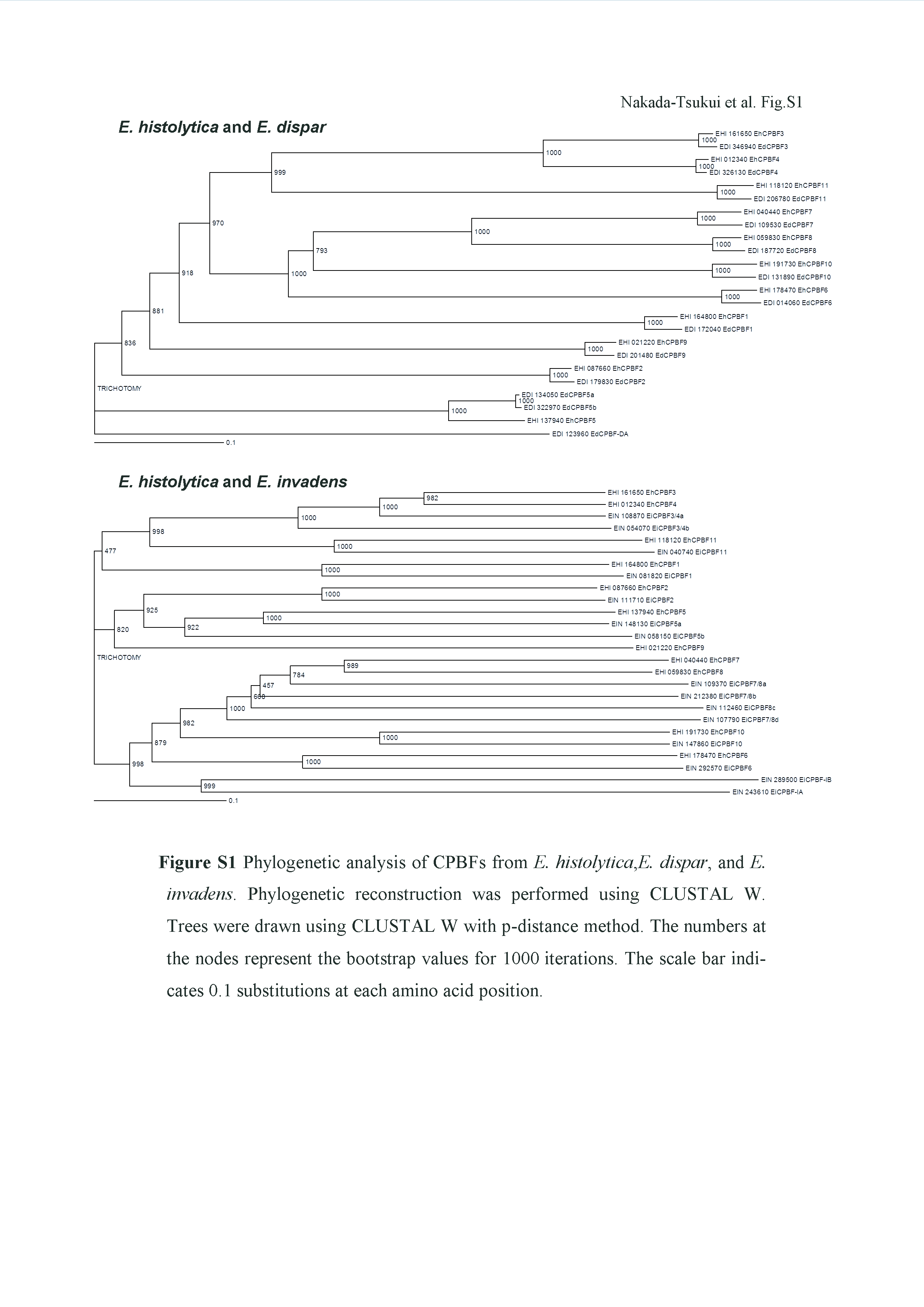

Supplement: Supplementary file 2 [file cmi0014-1299-SD1.tif]

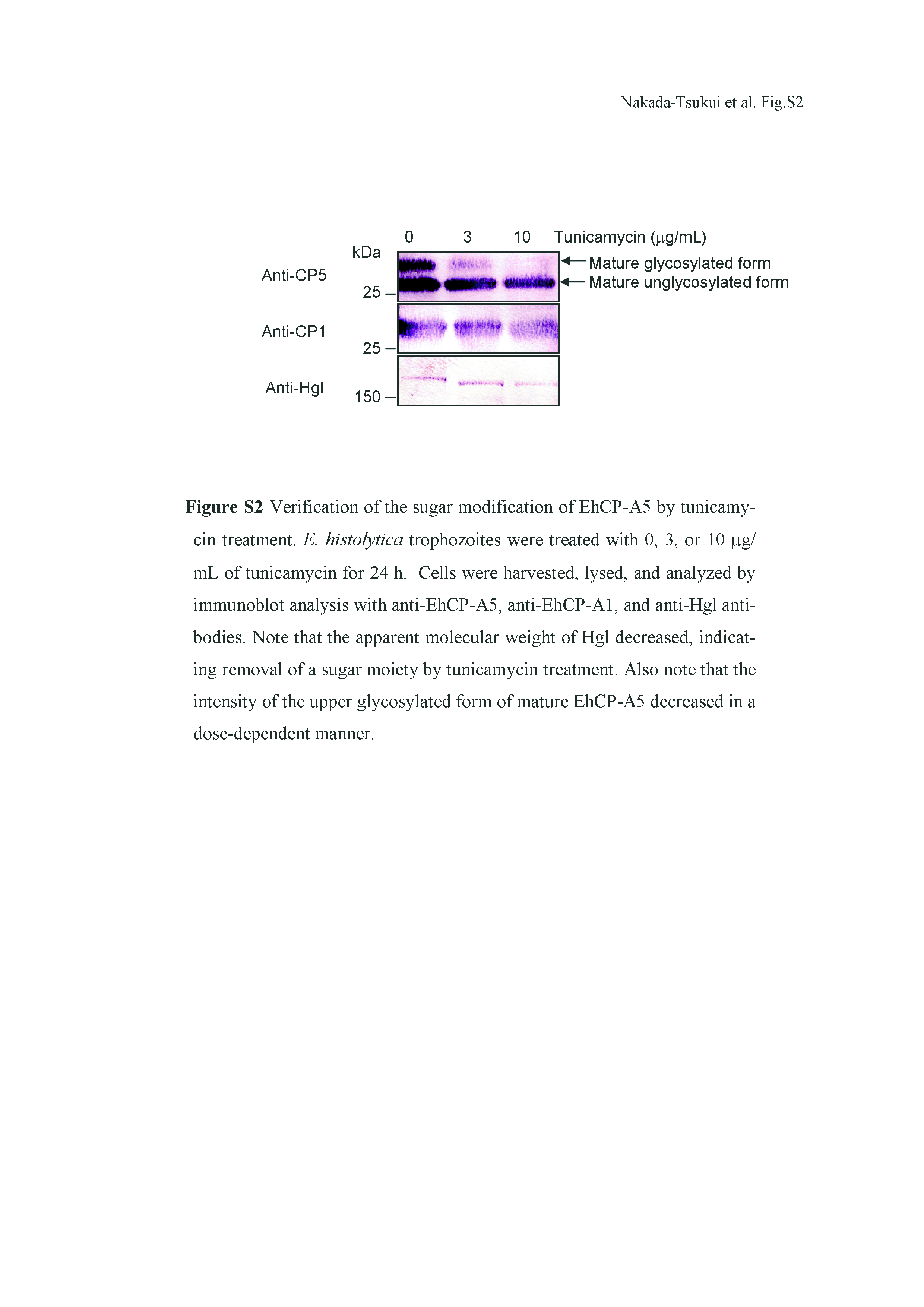

Supplement: Supplementary file 3 [file cmi0014-1299-SD2.tif]

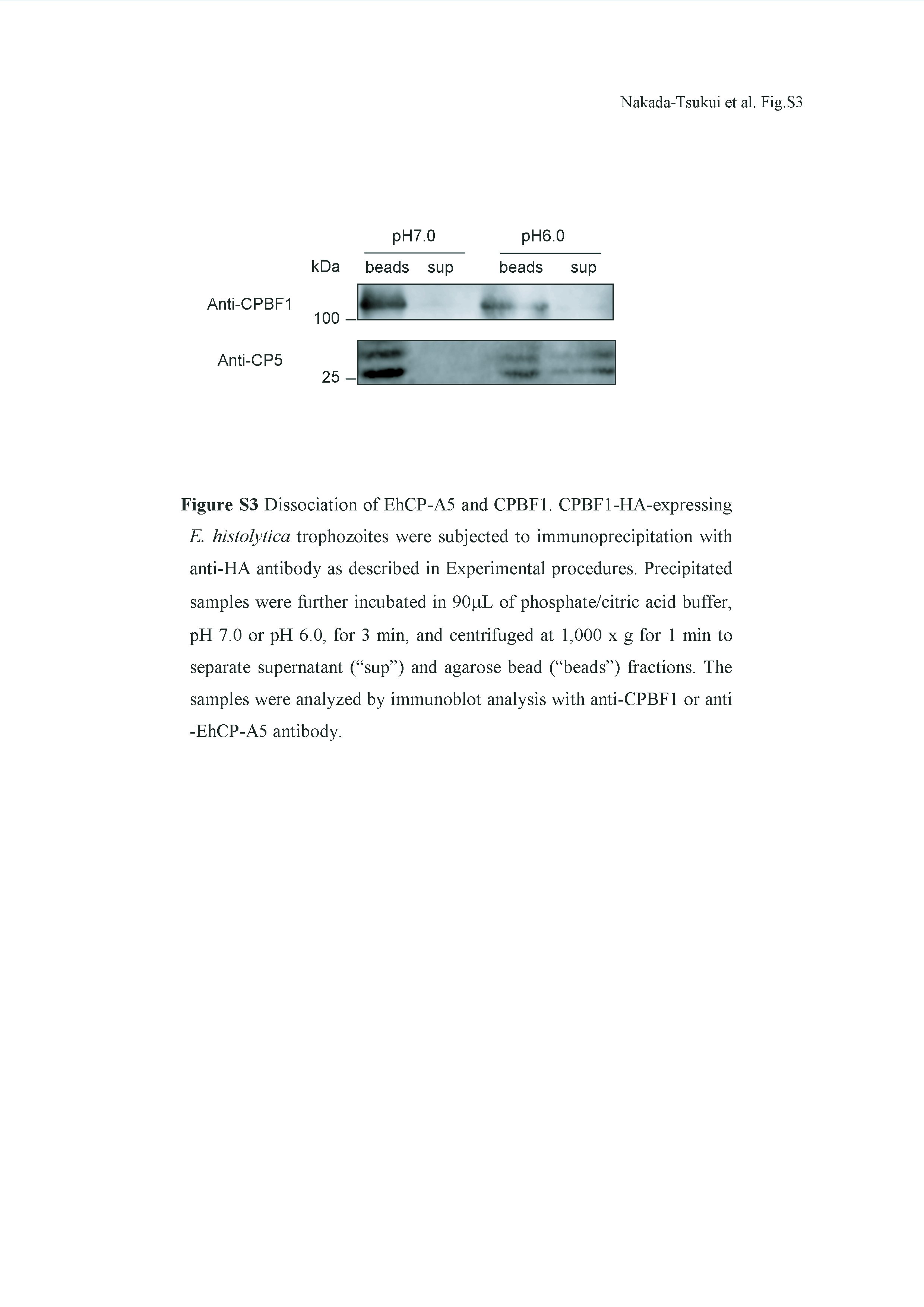

Supplement: Supplementary file 4 [file cmi0014-1299-SD3.tif]
